# Palbociclib-Induced Vitiligo-Like Lesions: A Report of a Challenging Case

**DOI:** 10.7759/cureus.62293

**Published:** 2024-06-13

**Authors:** Abdulmohsin K Algethami, Alhusain M Alshareef, Waseem K Alhawsawi, Bader A Bader, Arwa Alharbi, Raneem Alahmadi, Hanadi Alsatti, Amal H Abualola, Raneem Alqahtani

**Affiliations:** 1 Dermatology, King Abdulaziz Medical City, Jeddah, SAU; 2 Dermatology, King Fahad Military Medical Complex, Jeddah, SAU; 3 College of Medicine, Umm Al-Qura University, Makkah, SAU; 4 College of Medicine, King Saud Bin Abdulaziz University for Health Sciences, Jeddah, SAU; 5 Dermatology, King Saud Bin Abdulaziz University for Health Sciences, Jeddah, SAU

**Keywords:** dermatologic toxicity, vitiligo, breast cancer, cdk4/6 inhibitor, palbociclib

## Abstract

Cyclin-dependent kinase (CDK) 4 and 6 inhibitors, such as palbociclib, have emerged as essential in managing hormone receptor-positive (HR+), human epidermal growth factor receptor 2-negative (HER2−) advanced or metastatic breast cancer. While effective, these inhibitors can cause rare dermatologic side effects, including vitiligo-like depigmentation. We report a rare case of a 52-year-old female with HR+, HER2− metastatic breast cancer who developed vitiligo-like depigmentation following palbociclib treatment. The patient presented with asymptomatic depigmented lesions on the lower limbs and abdomen, appearing seven months after starting palbociclib. Examination and investigations confirmed the diagnosis after excluding other potential causes. Despite treatment with topical steroids and calcineurin inhibitors, there was no significant improvement, highlighting the need for more research into effective management strategies for drug-induced vitiligo. This case emphasizes the importance of recognizing rare dermatologic side effects of CDK4/6 inhibitors like palbociclib. Ongoing vigilance, reporting, and research are necessary to improve understanding and management of these side effects, ultimately enhancing patient care in oncology.

## Introduction

Breast cancer stands as the most diagnosed type of cancer among women globally and in Saudi Arabia [1]. Cyclin-dependent kinase (CDK) 4 and 6 inhibitors represent the latest approved class of targeted therapies for managing hormone receptor-positive (HR+) and human epidermal growth factor receptor 2-negative (HER2−) breast cancer [2]. The three approved CDK4/6 inhibitors (CDK4/6i) - palbociclib, ribociclib, and ademaciclib - are usually used in combination with endocrine therapy (fulvestrant, letrozole, exemestane, or anastrozole) which is the preferred approach for first-line and second-line management of hormone receptor-positive (HR+), HER2-negative, advanced, or metastatic breast cancer [2].

Generally, the CDK4/6 inhibitors are well tolerated and adverse reactions can be effectively managed with dose adjustment and appropriate care measures. Mild adverse events include neutropenia, diarrhea, and fatigue. Furthermore, CDK 4/6 inhibitors can also be associated with several potential dermatologic toxicities, and the most frequent dermatological adverse event is alopecia [2-4]. A case series reported that vitiligo can occur in patients treated with CDK4/6 inhibitors [3].

Vitiligo is a disease characterized by the appearance of white depigmented patches on the skin. It affects 0.1-2% of the world’s population; compared to other skin disorders, vitiligo has a significant negative psychological impact [5].

Vitiligo-like lesions induced by palbociclib are rare. To our knowledge, only four cases of palbociclib-induced vitiligo-like lesions have been reported in the literature [3,5,6].

Here we describe a rare case of a 52-year-old female, a known case of breast cancer, who came with depigmented lesions developed after treatment with palbociclib, a CDK4/6 inhibitor.

## Case presentation

**Figure 1:**
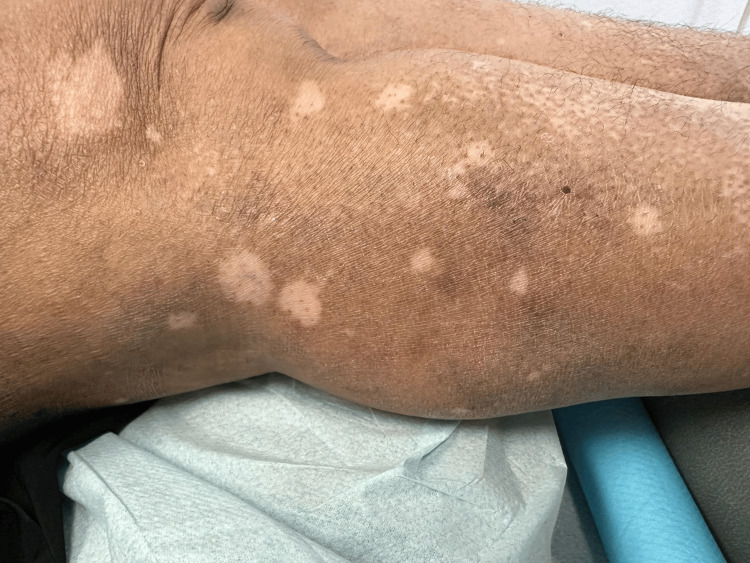
Over the right leg around the knee, there are multiple depigmented patches and depigmented macules

**Figure 2:**
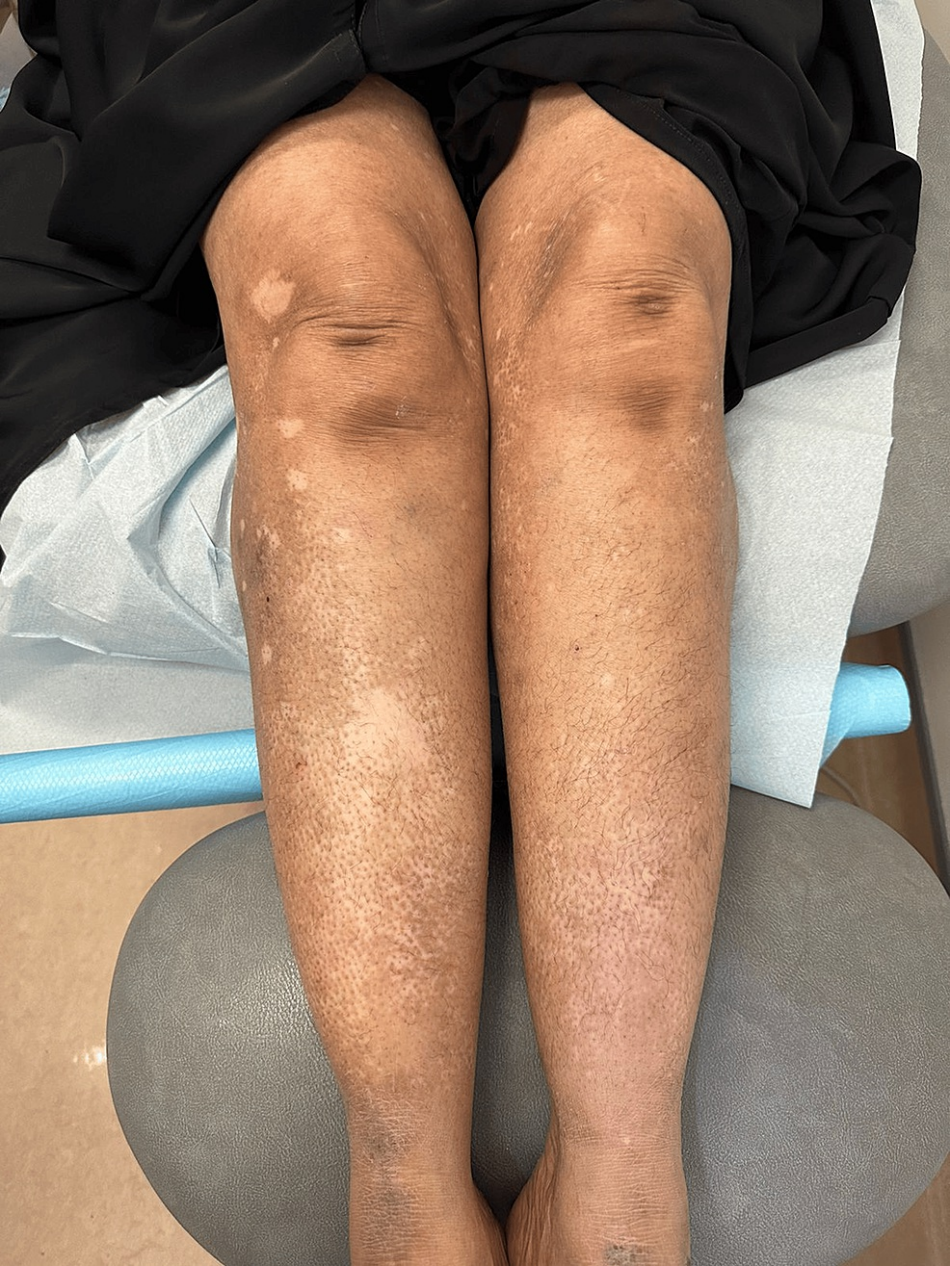
Over both legs, there are multiple scattered depigmented patches and depigmented macules

**Figure 3:**
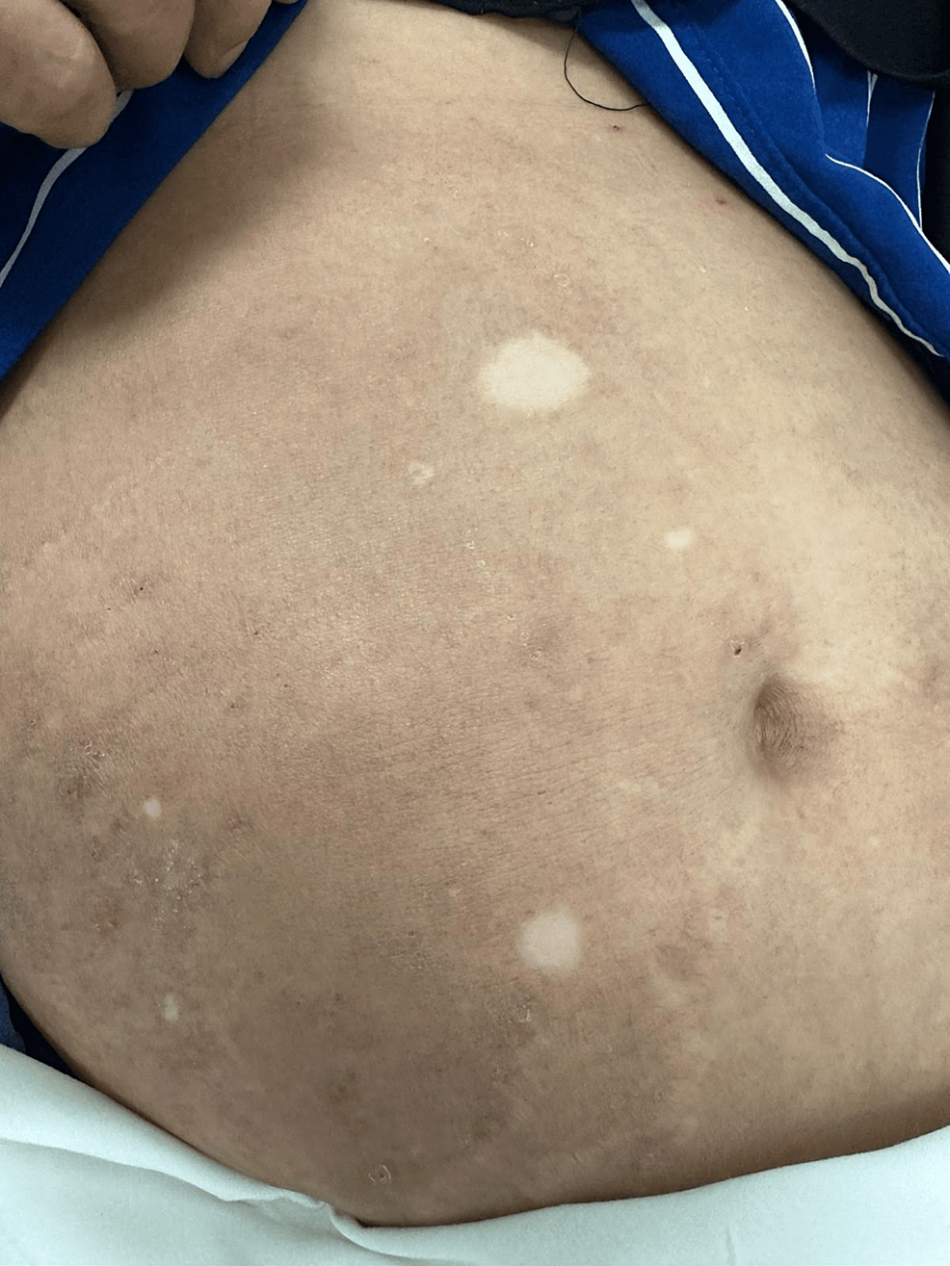
Over the abdomen, there are a few scattered depigmented patches

A 52-year-old female, a known case of right breast cancer with metastasis to bone diagnosed one year ago and on palbociclib, presented to the dermatology outpatient clinic with a complaint of asymptomatic depigmented skin lesions over bilateral lower limbs and abdomen for one month (Figures 1-3).

Further investigations and inquiry revealed that the patient has been receiving palbociclib 100 mg once a day for 21 days with seven days off continuously for seven months. In addition, he was switched on it after stopping abemacicib due to generalized severe itching and dryness. The lesions appeared all at the same time in the legs and the lower abdomen, and they were not progressing not preceded by erythema, starting after seven months of use of the medication. The patient denied any itchiness, discharge, or bleeding. Moreover, personal and family histories were negative in terms of vitiligo or other autoimmune disorders. Blood laboratory data showed no significant findings. The differential diagnosis for any depigmented lesions is subdivided into generalized depigmentation and localized depigmentation. Generalized depigmentation includes the following: chemical leukoderma, Vogt Koyanagi Harada disease, scleroderma leukoderma, chronic GVHD (graft-versus-host disease), chronic onchocerciasis, tertiary pinta, hypopigmented mycosis fungoides, and melanoma-associated leukoderma. On the other hand, localized depigmentation includes the following: post-inflammatory pigmentations, sarcoidosis, periorbital discoid lupus, poliosis, lichen sclerosus, and extramammary Paget's disease. A diagnosis of palbociclib-induced vitiligo-like lesions was made after excluding all these differential diagnoses. The absence of a personal or family history of autoimmune disorders, along with the temporal association with palbociclib therapy, was pivotal in ascertaining the diagnosis of drug-induced vitiligo in our patient. On examination, the patient was vitally stable and had a BMI of 25.6. On general inspection, the patient was conscious, oriented, and not distressed. Skin examination revealed multiple de-pigmented skin lesions located in the bilateral lower extremities and abdomen comprising 3% of body surface area. No scales, erythema, or secondary changes were appreciated. No hair, nail, or mucosal involvement was there. A woods lamp examination was performed on the patient, which showed chalky white fluorescence confirming the diagnosis. The treatment plan was discussed with the patient, and topical steroids were prescribed twice daily for two weeks then twice weekly and topical tacrolimus five times weekly use. There was no improvement seen after a six-month follow-up.

## Discussion

Palbociclib, a cyclin-dependent kinase 4 and 6 inhibitor (CDK4/6i), has emerged as a cornerstone in the treatment of HR+, HER2− advanced or metastatic breast cancer, offering a novel mechanism of action by interrupting cell cycle progression and thereby inhibiting tumor proliferation [2]. While its efficacy has been widely recognized, the spectrum of its adverse effects, particularly dermatologic ones, is still being delineated. Among these, vitiligo-like depigmentation is an exceedingly rare yet psychologically impactful side effect, with our case adding to the scant literature on this phenomenon [4]. The pathogenesis of palbociclib-induced vitiligo remains speculative, yet it might be hypothesized that CDK4/6 inhibition affects melanocyte proliferation or survival, given their dependence on the CDK4/6 pathway for cell cycle progression [5,7]. The temporal correlation between the initiation of palbociclib and the onset of depigmented lesions in our patient underscores a probable causal relationship. However, given the rarity of such adverse effects, it also prompts consideration of underlying predispositions, possibly genetic, that could render certain individuals more susceptible to this effect [4].

The clinical management of palbociclib-induced vitiligo involves a multidisciplinary approach, considering the cosmetic concerns and potential psychological impact associated with vitiligo. The use of topical steroids and calcineurin inhibitors, as in our case, aligns with first-line treatments for vitiligo, aiming to promote repigmentation through anti-inflammatory mechanisms and melanocyte stimulation [4]. However, the effectiveness of these treatments in the context of drug-induced vitiligo is less clear, warranting further investigation [3].

The rarity of palbociclib-induced vitiligo calls for continued vigilance and reporting of such adverse effects to better understand their incidence, pathogenesis, and optimal management strategies. It also underscores the need for patient education and counseling regarding the potential adverse effects of new cancer therapies, ensuring that patients are prepared for and supported in managing these effects should they occur.

## Conclusions

In conclusion, as the application of CDK4/6 inhibitors expands in the realm of breast cancer therapy, the spectrum of associated adverse effects will likely broaden, necessitating ongoing research and awareness. This case of palbociclib-induced vitiligo not only contributes to the dermatologic toxicity profile of CDK4/6 inhibitors but also emphasizes the need for awareness and research on rare side effects to improve patient care and outcomes in oncology. We recommend more research be conducted to document causal relationships between vitiligo and palbociclib.
